# Ultrasound of entheses in ankylosing spondylitis patients: The importance of the calcaneal and quadriceps entheses for differentiating patients from healthy individuals

**DOI:** 10.6061/clinics/2019/e727

**Published:** 2019-04-04

**Authors:** Suellen Narimatsu Ishida, Rita Nely Vilar Furtado, André Rosenfeld, Jorge Ernesto Passos Proglhof, Germana Brigida Queiroga Estrela, Jamil Natour

**Affiliations:** I Disciplina de Reumatologia, Escola Paulista de Medicina, Universidade Federal de Sao Paulo, Sao Paulo, SP, BR; II Departamento de Diagnostico por Imagem, Escola Paulista de Medicina, Universidade Federal de Sao Paulo, Sao Paulo, SP, BR

**Keywords:** Entheses, Ultrasound, US, Ankylosing Spondylitis, Healthy Individual

## Abstract

**OBJECTIVES:**

To compare the ultrasonographic findings of entheses in ankylosing spondylitis (AS) patients with those of healthy control individuals and to assess the ability of ultrasound (US) to identify aspects related to the disease.

**METHODS:**

A cross-sectional study involving 50 patients with AS and 30 healthy controls was performed. Clinical assessment included the use of a visual analog scale for pain, assessment of swelling of the enthesis, global assessments for patients and physician, use of a disease activity index, mobility and dysfunctional indices, erythrocyte sedimentation rate and clinical enthesitis index. US was performed for the following entheses by two experienced musculoskeletal radiologists: brachial triceps, distal quadriceps, proximal and distal patellar tendons, calcaneal tendon, and plantar fascia; the total and subitems of the Madrid Sonographic Enthesitis Index were used for evaluations.

**RESULTS:**

Comparison between groups showed a statistically significant difference with worse scores in AS patients, with bone erosion of the calcaneal enthesis and bone erosion and thickening of the plantar fascia. The odds ratio for thickening of the plantar fascia in the AS group was 3.47, according to logistic regression analysis. The AS group also had worse scores regarding the presence of calcification in the quadriceps enthesis, with a fivefold increased risk.

**CONCLUSION:**

US analysis showed that only entheses of the foot and quadriceps were able to differentiate AS patients from healthy individuals.

## INTRODUCTION

Spondyloarthritis is an inflammatory disease that can affect the axial and peripheral skeleton. It has been shown that early diagnosis of this disease is difficult because of low specificity and fluctuating symptoms (1). Ankylosing spondylitis (AS) is the main type of spondyloarthritic disease (2). A Brazilian multicenter study with 1,505 spondyloarthritis patients showed that 65.4% of patients had AS (3).

Enthesitis is inflammation of the enthesis, which is the site where the tendons or ligaments insert into the bone (4), and it is considered the hallmark of spondyloarthritis (5,6). Enthesitis often occurs in the lower limbs, which can lead to structural damage, such as tendon injury, enthesophyte formation and dysfunction. Enthesophyte formation is considered a late finding of spondyloarthritis and chronic enthesitis (7). However, some authors have demonstrated a high prevalence of subclinical enthesitis using ultrasound (US) (8,9).

Complementary tests can help to diagnose and monitor patients. Currently, conventional radiography is still widely used as part of the classical classification criteria for spondyloarthritis, the modified New York criteria (10) and the Assessment of SpondyloArthritis International Society (ASAS) Group criteria (11). However, radiography has low sensitivity for initial inflammatory changes compared with magnetic resonance imaging (MRI) and US (12). MRI is a more sensitive method than radiography and tomography because this modality can be used to visualize bone edema before radiographic changes occur (13); however, this is a high-cost and low-availability method when compared with US (14). Because of the delay in the onset of radiographic lesions, a delay of up to 7 years in the diagnosis of spondyloarthritis can occur (15).

Many authors have demonstrated the superiority of US in relation to clinical examinations for detecting changes in entheses (8,16,17). Some authors have noted that the agreement for detecting changes between power Doppler (PD) US (PDUS) and MRI is at least good (18,19) and that the rates and interobserver agreement of US are good and high for evaluating hypoechogenicity, thickening, calcification, enthesophyte formation, bone erosion, and PD (20).

PD is a complementary tool to US that is used with grayscale to increase the sensitivity for detecting inflammatory changes (16,17). Despite evidence supporting the use of PDUS for the diagnosis of subclinical inflammation (8,16,17) and follow-up treatment of patients with spondyloarthritis (21), there is still a lack of standardized definitions of enthesitis and ultrasonographic parameters, which may contribute to failures in studies and make data comparisons difficult (1).

Few studies involving healthy participants have been published in this context. We believe that comparison of US findings from patients with inflammatory disease and healthy control individuals can help to identify aspects of the disease, which could help in diagnosis.

The aim of this study was to compare US findings of entheses between patients with AS and healthy controls based on the Madrid Sonographic Enthesitis Index (MASEI) (17) and to assess the ability of US to identify aspects related to AS.

## MATERIALS AND METHODS

A cross-sectional study involving 50 AS patients classified by the modified New York criteria (10) or ASAS criteria (11) and 30 healthy individuals was performed. Patients were recruited from rheumatology outpatient clinics at the university, and the healthy participants were blood donors from the blood bank of the institution or university staff, family, and friends. Individuals with a history of litigation, uncontrolled fibromyalgia, intraarticular corticosteroid injections in the last 3 months, previous surgery involving the studied structure, peripheral neuropathy, diabetes mellitus, hypothyroidism and venous insufficiency of the lower limbs with edema and/or ochre dermatitis were excluded.

### Clinical Assessment

Healthy control individuals completed a questionnaire with demographic data, such as weight, height, and age, and they underwent a US evaluation. For patients with AS, apart from the US assessment, a clinical evaluation was performed by a rheumatologist who was “blinded” to the ultrasonographic findings. The assessment consisted of the use of a visual analog scale (VAS; from 0–10 cm) for evaluating pain and swelling, physician and patient global assessment, history of medication use and doses, and analysis of the erythrocyte sedimentation rate (ESR) during the first hour. The rheumatologist also administered the following questionnaires regarding disease and clinical indices: the clinical index of peripheral enthesitis from the Spondyloarthritis Research Consortium of Canada (SPARCC) (22); a disease activity index developed by the ASAS using the ESR (ASDAS-ESR) (23); and the Bath Ankylosing Spondylitis Disease Activity Index (BASDAI) (24). Other questionnaires pertaining to functionality and metrology (questionnaires validated for Portuguese) included the Bath Ankylosing Spondylitis Functional Index (BASFI) (25), Bath Ankylosing Spondylitis Metrology Index (BASMI) (26) and Health Assessment Questionnaire for Spondyloarthropathies (HAQ-S) (27).

### US Assessment

Two radiologists with 10 years of experience conducting musculoskeletal US examinations performed these assessments and were “blinded” to the group of the participants and their clinical assessment findings. A MyLab60 Xvision (Esaote Biomedica, Genova, Italy) equipped with a linear transducer with a frequency of 6–18 MHz was used. The following entheses were assessed bilaterally: brachial triceps, distal quadriceps, proximal patellar tendon (in the lower pole of the patella), distal patellar tendon (tibial tuberosity), calcaneal tendon, and plantar fascia. Fifty patients and 30 healthy control subjects underwent US examinations, for a total of 160 entheses analyzed at each site.

**Figure 1:**
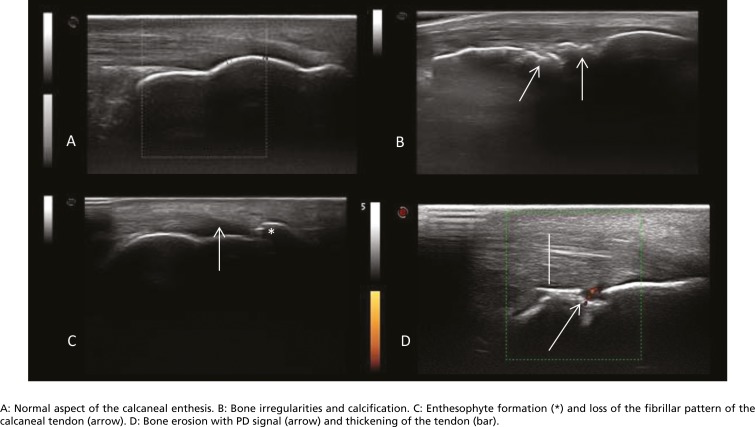
Ultrasound images of the calcaneal enthesis.

The US assessment of the enthesitis was based on the MASEI (17), which assesses the presence of calcification or ossification, bursitis, bone erosion, PD signal, pathologic structural change and thickening of the tendon at the site of insertion. *Calcification* was graded from 0–3: 1, small calcification or ossification with an irregularity in cortical bone; 2, clear presence of enthesophyte formation (hyperechoic spurs forming at a tendon insertion into bone and growing in the direction of the natural pull of the tendon involved), or medium-sized calcifications or ossifications; and 3, large calcifications or ossifications. *Bursitis* was defined as an area compatible with the site of a bursa with hypoechoic or anechoic contents that were compressible with the transducer at the calcaneal and distal patellar entheses. *Bone erosion* was defined as cortical breakage with bone contour defects in 2 perpendicular planes. *PD signal* indicates flow with capture of small vessels using a repeated pulse frequency of 500 Hz, and the gain was the largest possible after removal of artifacts. *Pathologic structural change* occurred if there was loss of a fibrillar pattern, hypoechoic appearance or fusiform thickening. *Thickening of the tendon* insertion was determined with the use of the following measures: brachial triceps greater than 4.3 mm, quadriceps greater than 6.1 mm, patellar tendon greater than 4.0 mm, calcaneus greater than 5.29 mm, and plantar fascia greater than 4.4 mm (Figure 1) (17).

The images obtained by one of the radiologists were recorded, and analysis of interobserver agreement was performed at a different time by another ultrasonographer in 10% of the total patient sample.

### Statistical Analysis

For statistical analysis, we used SPSS for Windows (version 17.0). Numerical data are described as the mean and standard deviation. Student's *t*-test was applied to compare numerical parametric variables. The Mann–Whitney test was applied to compare numerical nonparametric variables. The *χ* ^2^ test was used to compare categorical variables between groups. Logistic regression analysis was used to calculate the odds ratios (ORs) between groups. Receiver operating characteristic (ROC) curve analysis was used to identify individuals with AS. To analyze interobserver agreement, we used the Cohen Kappa (κ) coefficient of concordance for categorical data and the intraclass correlation coefficient (ICC) for continuous data with a 95% confidence interval (95% CI). Statistical significance was considered at 5% (*p*<0.05).

### Ethics

The study was approved by the Ethics Committee of the institution, Universidade Federal de São Paulo – UNIFESP – Hospital São Paulo (submission number 1649/09), and was performed in accordance with the Helsinki Declaration of 1975, which was revised in 1983. All participants were between 18 and 65 years of age, and they signed an informed consent form.

## RESULTS

The average age of patients in the AS group (ASG) was slightly higher than that in the control group (CG). There was no significant difference in the demographic data, such as weight, height, body mass index and gender, between the groups (Table 1).

**Table 1:** Demographic and clinical characteristics of the sample.

		ASG N=50	CG N=30	*p*
Age (y) – mean (SD)		43.44 (9.91)	38.70 (8.52)	0.032*
Gender				0.724**
	M - n (%)	40 (80.00)	23 (76.70)	
	F – n (%)	10 (20.00)	7 (23.3)	
Weight (kg) – mean (SD)		71.89 (15.38)	75.35 (14.30)	0.413***
Height (m) – mean (SD)		1.66 (0.09)	1.68 (0.079)	0.348*
BMI		25.20 (4.93)	26.92 (10.01)	0.358***
MASEI – mean (SD)		16.32 (11.22)	10.70 (5.27)	0.063***
Disease duration (y)		11.11 (6.76)	-	-
Patient Global Assessment – VAS 0–10 cm		4.46 (2.55)	-	-
Physician Global Assessment – VAS 0–10 cm		3.22 (1.91)	-	-
BASDAI		3.19 (2.10)	-	-
BASFI		4.27 (2.67)	-	-
BASMI		4.74 (2.35)	-	-
HAQ-S		0.57 (0.49)	-	-
ASDAS-ESR		2.37 (0.99)	-	-
SPARCC		2.80 (3.87)	-	-
MASEI		16.32 (11.22)	-	-
ESR (mm)		19.94 (16.57)	-	-

ASG: ankylosing spondylitis group; CG: control group; Y: years; M: male; F: female; BMI: body mass index; VAS: visual analog scale; BASDAI: Bath Ankylosing Spondylitis Disease Activity Index; BASFI: Bath Ankylosing Spondylitis Functional Index; BASMI: Bath Ankylosing Spondylitis Metrology Index; HAQ-S: Health Assessment Questionnaire for Spondyloarthropathies; ASDAS-ESR: disease activity index developed by the Assessment of SpondyloArthritis International Society (using the ESR); SPARCC: Clinical index of peripheral enthesitis from the Spondyloarthritis Research Consortium of Canada; MASEI: Madrid Sonographic Enthesis Index; ESR: *erythrocyte sedimentation rate* in the first hour.

* Student's *t*-test;

** χ^2^ test;

*** Mann–Whitney test.

Patients in the ASG were assessed regarding related disease characteristics (Table 1) and drugs used (Table 2). The mean duration of the disease was 11.11 (±6.76) years, showing long-standing disease. An estimated 26% of patients were receiving continuous treatment with nonsteroidal anti-inflammatory drugs (NSAIDs), and 50% were receiving immunobiological treatment with antitumor necrosis factor-alpha (anti-TNFα) drugs.

**Table 2:** Drugs used in the Ankylosing Spondylitis Group.

		N (%)	Minimum	Maximum	Mean	Standard Deviation
TIME OF USE
	NSAIDs (mo)	13 (26)	1.00	180.00	35.69	55.59
	Corticosteroids (mo)	1 (2)	6.00	6.00	6.00	-
	Methotrexate (mo)	6 (12)	1.00	120.00	38.33	47.93
	Sulfasalazine (mo)	6 (12)	2.00	120.00	31.83	44.92
	Infliximab (mo)	10 (20)	12.00	72.00	39.30	19.72
	Etanercept (mo)	13 (26)	2.00	36.00	17.61	13.20
	Adalimumab (mo)	2 (4)	24.00	48.00	36.00	16.97
	Joint injections (n)	21 (84)	0.00	3.00	0.70	0.93
DOSAGE
	Diclofenac (mg/day)	2 (4)	100.00	100.00	100.00	-
	Nimesulide (mg/day)	1 (2)	200.00	200.00	200.00	-
	Naproxen (mg/day)	6 (12)	500.00	1,500.00	958.33	332.29
	Indomethacin (mg/day)	4 (8)	50.00	100.00	81.25	23.93
	Methotrexate (mg/wk)	6 (12)	12.50	20.00	16.66	3.76
	Sulfasalazine (mg/day)	6 (12)	1,000.00	3,000.00	1,583.00	735.98
	Prednisolone (mg/day)	1 (2)	5.00	5.00	5.00	-

mo: months; NSAID: Nonsteroidal anti-inflammatory drug; wk: weeks.

The average BASDAI score was 3.19 (±2.10), showing that the studied population was below the index of intense activity (<4). Regarding dysfunction, the BASFI index and HAQ-S showed moderate dysfunction with values of 4.27 (±2.67) and 0.57 (±0.49), respectively. The ESRs ranged widely, from 1 mm to 82 mm, and the average was 19.94 (±16.57) mm. The mean ASDAS-ESR was 2.37 (±0.99), indicating moderate to high disease activity (23) (Table 1).

US comparisons were performed in 100 entheses of patients in the ASG and in 60 entheses of patients in the CG at six different sites (Table 3). A total of 960 entheses were assessed in this study. US parameters that showed significant differences between the groups, with worse scores in the ASG with regard to bone erosion in the calcaneal enthesis (*p*<0.001), bone erosion of the plantar fascia (*p*=0.046), thickening of the plantar fascia (*p*=0.002), and calcification of the distal quadriceps (*p*=0.001).

**Table 3:** Comparison of ultrasonographic findings of the entheses between groups.

Enthesis		ASG N=100 n (%)	CG N=60 n (%)	*p* (χ^2^)	OR
Calcaneal (Achilles) tendon
	Calcification			0.097	
	0	52 (52)	30 (50.0)		
	1	13 (13)	6 (10.0)		
	2	25 (25)	23 (38.3)		
	3	10 (10)	1 (1.7)		
	Bursitis	10 (10)	5 (8.3)	0.787	
	Erosion	17 (17)	0	<0.001	
	Power Doppler signal	6 (6)	0	0.084	
	Thickening	36 (36)	15 (25.0)	0.148	
	Structural change	17 (17)	5 (8.3)	0.123	
Plantar fascia
	Calcification			0.296	
	0	69 (69)	44 (73.3)		
	1	12 (12)	3 (5.0)		
	2	17 (17)	13 (21.7)		
	3	2 (2)	0		
	Erosion	7 (7)	0	0.040	
	Power Doppler signal	0	0	1.000	
	Thickening	38 (38)	9 (15.0)	0.002	3.473 (0.003)
	Structural change	27 (27)	10 (16.7)	0.133	
Proximal patellar tendon
	Calcification			0.470	
	0	95 (95)	59 (98.3)		
	1	3 (3)	1 (1.7)		
	2	2 (2)	0		
	3	0	0		
	Erosion	0	0	1.000	
	Power Doppler signal	0	0	1.000	
	Thickening	61 (61)	33 (55)	0.455	
	Structural change	3 (3)	3 (5.0)	0.519	
Distal patellar tendon
	Calcification			0.822	
	0	88 (88)	52 (86.7)		
	1	9 (9)	6 (10.0)		
	2	2 (2)	2 (3.3)		
	3	1 (1)	0		
	Bursitis	6 (6)	2 (3.3)	0.711	
	Erosion	2 (2)	0	0.528	
	Power Doppler signal	1 (1)	0	1.000	
	Thickening	69 (69)	37 (61.7)	0.342	
	Structural change	8 (8)	9 (15.0)	0.164	
Quadriceps femoralis tendon
	Calcification			0.001	5.262 (0.000)
	0	59 (59)	53 (88.3)		
	1	14 (14)	3 (5.0)		
	2	16 (16)	4 (6.75)		
	3	11 (11)	0		
	Erosion	2 (2)	0	0.528	
	Power Doppler signal	1 (1)	0	1.000	
	Thickening	45 (45)	23 (38.3)	0.409	
	Structural change	14 (14)	6 (10.0)	0.459	
Triceps Brachii	Calcification			0.337	
	0	72 (72)	50 (83.3)		
	1	10 (10)	5 (8.3)		
	2	16 (16)	4 (6.7)		
	3	2 (2)	1 (1.7)		
	Erosion	0	1 (1.7)	0.375	
	Power Doppler signal	0	0	1.000	
	Thickening	70 (70)	41 (68.3)	0.825	
	Structural change	7 (7)	2 (3.3)	0.331	

ASG: ankylosing spondylitis group; CG: control group; OR: odds ratio for belonging to the AS group. The χ^2^ and Fisher tests were used.

The logistic regression analysis identified the odds of belonging to the ASG from two US measurements. Regarding thickening of the plantar fascia, logistic regression analysis showed an OR of 3.47 (*p*=0.003) in the ASG (Table 3). A significant difference was also observed between groups, with worse scores for the ASG than for the CG for the presence of calcification in the quadriceps enthesis (*p*=0.001), with an OR of 5.26 (*p*<0.001).

The average total MASEI score was not different between groups (ASG, 16.32±11.22 vs. CG, 10.70 ±8.52; *p*=0.063). The area under the ROC curve (AUC) was not able to determine the accuracy of the total MASEI score for predicting the diagnosis of enthesitis by AS (0.625±0.062; *p*=0.064).

Interobserver agreement for quantitative data regarding tendon thickness was excellent for all tendons, ranging from 0.968 for the triceps (95% CI, 0.909–0.989) to 0.990 for the calcaneus by the ICC (28) (95% CI, 0.970–0.997) (Table 4). In the semiquantitative analysis, the level of agreement among the raters for bursitis, erosions, and PD was 100% (κ=1.00). Regarding calcification, the triceps yielded the lowest value (0.75), showing good agreement; for the other tendons, agreement was excellent, ranging from 0.89 (quadriceps) to 1.00. Structural changes showed 100% agreement in the analysis of tendons, except for the quadriceps, which was weak (κ=0.385). For tendon thickness, the κ index ranged from moderate agreement for the triceps (κ=0.600) to excellent agreement for the quadriceps and plantar fascia (95% CI, 0.818–1.00) (Table 5).

**Table 4:** Concordance indices for quantitative variables (US Evaluation of Tendon Thickness) for Assessing Interobserver Agreement.

Tendon	ICC (*p*)	Minimum and Maximum
Brachial triceps	0.968 (0.000)	0.909 to 0.989
Distal quadriceps	0.971 (0.000)	0.917 to 0.990
Proximal patellar tendon	0.987 (0.000)	0.961 to 0.995
Distal patellar tendon	0.975 (0.000)	0.789 to 0.994
Achilles tendon	0.990 (0.000)	0.970 to 0.997
Plantar fascia	0.970 (0.000)	0.916 to 0.990

ICC: Intraclass correlation coefficient.

**Table 5:** Cohen's Kappa Correlation for Semiquantitative Measures in the Interobserver Analysis.

Ultrasound variable	Enthesis	Cohen's Kappa (*p*)
Calcification	Triceps brachii	0.750 (0.000)
	Quadriceps femoralis	0.895 (0.000)
	Proximal patellar tendon	1.000 (0.000)
	Distal patellar tendon	1.000 (0.000)
	Calcaneal or Achilles	1.000 (0.000)
	Plantar fascia	1.000 (0.000)
Bursitis	Distal patellar tendon	1.000 (0.000)
	Calcaneal	1.000 (0.000)
Bone erosion	Triceps brachii	1.000 (0.000)
	Quadriceps femoralis	1.000 (0.000)
	Proximal patellar tendon	1.000 (0.000)
	Distal patellar tendon	1.000 (0.000)
	Calcaneal or Achilles	1.000 (0.000)
	Plantar fascia	1.000 (0.000)
Power Doppler signal	Triceps brachii	1.000 (0.000)
	Quadriceps femoralis	1.000 (0.000)
	Proximal patellar tendon	1.000 (0.000)
	Distal patellar tendon	1.000 (0.000)
	Calcaneal or Achilles	1.000 (0.000)
	Plantar fascia	1.000 (0.000)
Structural change	Triceps brachii	1.000 (0.000)
	Quadriceps femoralis	0.385 (0.051)
	Proximal patellar tendon	1.000 (0.000)
	Distal patellar tendon	1.000 (0.000)
	Calcaneal or Achilles	1.000 (0.000)
	Plantar fascia	1.000 (0.000)
Thickening	Triceps brachii	0.600 (0.009)
	Quadriceps femoralis	0.818 (0.001)
	Proximal patellar tendon	1.000 (0.000)
	Distal patellar tendon	0.862 (0.000)
	Calcaneal or Achilles	0.862 (0.000)
	Plantar fascia	1.000 (0.000)

## DISCUSSION

US in rheumatology has been widely used in recent decades to aid in the diagnosis and follow-up treatment of rheumatic diseases, given the greater sensitivity of US than clinical examination (8,9,16,17).

In our study, the comparison between findings on US for enthesitis in patients with diagnosed AS and healthy control participants was significantly different in the analysis for bone erosions in the calcaneal tendon enthesis (*p*<0.001) and in the plantar fascia (*p*=0.046), for thickening of the plantar fascia (*p*=0.002), and for calcification in the quadriceps femoris (*p*=0.001). According to logistic regression analysis, it was possible to calculate the OR for belonging to the ASG for plantar fascial thickening (a value of 3.47) and for enthesophyte formation and calcification in the quadriceps femoris (OR=5.26).

A high frequency of enthesitis of the lower limbs was found, especially in the calcaneus and plantar fascia, reinforcing previous findings (8,16,17). The sites of the enthesitis associated with spondyloarthritis that were most affected by changes on US in these previous studies were the calcaneal tendon and plantar fascia, which were the same as those that were altered in this study.

According to de Miguel et al. (17), a comparison of patients with spondyloarthritis or AS with healthy subjects showed a significant difference in MASEI scores between the groups. Although we studied a larger number of participants, the difference between the mean MASEI scores was not statistically significant between our groups. This result may be due to a very large difference between the disease duration of our patients, ranging from 1.5–40 years of disease. The inclusion of patients with early and long-standing disease could be a cause for this disagreement between studies. A study conducted with a larger sample with groups featuring varying durations of disease could help.

PDUS seems to be an important and promising tool for the evaluation of enthesitis. However, PDUS still lacks a consensus on the definitions of enthesitis using US to support the method as a reference for the diagnosis and treatment of patients with peripheral spondyloarthropathies (1).

Two studies have highlighted the use of PD in inflammatory disease to diagnose enthesitis. As part of the MASEI, bone erosion and PD showed better predictive value for inflammatory enthesitis in affected patients than in control participants (17). In 2011, D'Agostino et al. (29) demonstrated high predictive value of PD for the diagnosis of spondyloarthritis when patients with clinical suspicion were evaluated.

The fact that all patients were receiving treatment for at least 1.5 years (and many of them for several years) could have influenced the low prevalence of PD signals or active inflammation. In our study, we identified PD signals in only 8 entheses (1.3%), in contrast to previous data obtained by D'Agostino et al. (16), who found a PD signal in 81% of evaluated entheses, and de Miguel et al. (17), who found 60% positivity. The low prevalence of PD signals was also observed by Spadaro et al. (30), who found these signals in only 5% of participants; similar to our data, 58% of patients were using anti-TNFα drugs, which could have influenced the low vascularization in entheses because these drugs are responsible for blocking an important inflammatory pathway.

Other authors, who assessed PDUS in heels using MRI as the reference standard, reported no specific findings of inflammatory disease in affected patients compared with control participants without inflammatory disease (31). Furthermore, during the evaluation of painful heels in patients with spondyloarthritis stemming from a traumatic injury, no findings were specific for spondyloarthritis (19). These data contradict our findings, which identified bone erosion and PD signals only in the calcaneal entheses of patients.

Calcification or enthesophyte formation in the quadriceps enthesis was presented as a risk factor for AS, indicating a five times higher risk of a lesion due to inflammatory disease. This was the first study to identify the importance of the quadriceps enthesis for differentiating patients with AS from healthy subjects. The changes described are considered by some authors to indicate chronic enthesitis (1,7) and might be related to long-standing disease. While this finding is not helpful for the early diagnosis of the disease, it could help in cases of diagnostic doubt and in classifying patients. Therefore, we believe that studies that help to standardize which locations are used to evaluate AS, as well as which lesions should be considered, will be valuable and should be encouraged. We also believe that the quadriceps enthesis should receive greater attention in future evaluations, given the possibility that this enthesis can be used to help identify characteristic lesions of AS and assist in diagnosis.

Our study had limitations. The number of patients and control individuals assessed, although calculated, may have been insufficient to demonstrate differences in US evaluation findings between the groups. It was not possible to perform logistic regression analysis between the groups for the bone erosion in the calcaneus and plantar fascia. In both cases, no healthy individuals had these lesions, making application of a statistical test impossible.

The present study demonstrated that, when applied by trained evaluators, PDUS may have good to excellent interobserver agreement. Therefore, US can be a very useful tool for assessing entheses in patients with AS because this modality is a safe imaging method that requires no radiation exposure or contrast material; furthermore, US is fast to perform and inexpensive when compared with MRI.

The presence of US findings (bone erosions) in the heel (entheses of the calcaneal tendon and plantar fascia) was an important characteristic that differentiated patients with long-standing AS from healthy controls. However, increased thickness of the plantar fascia and calcification of the quadriceps enthesis in our study were the only parameters that showed an OR that identified patients as belonging to the ASG, and we believe that analysis of these specific entheses should be performed more than it is currently.

We conclude that bone erosion in the calcaneal enthesis (tendons and fascia), thickening of the plantar fascia, and, calcification in the quadriceps enthesis are US findings that can differentiate patients with AS from healthy controls. The total MASEI score was not able to differentiate patients with AS from healthy individuals in this study.
